# Case Report: A case of micropapillary thyroid carcinoma with level I cervical lymph node metastasis

**DOI:** 10.3389/fonc.2025.1650616

**Published:** 2025-09-04

**Authors:** Qi Yang, Wei Cheng, Qin-long Liang, Li Yan

**Affiliations:** ^1^ Head and Neck Oncology, Shaanxi Provincial Cancer Hospital, Xi’an, China; ^2^ Department of Blood Transfusion, Shaanxi Provincial Cancer Hospital, Xi’an, China

**Keywords:** thyroid carcinoma, thyroid papillary carcinoma, level I cervical lymph node, lymph node metastasis, neck

## Abstract

Papillary thyroid carcinoma frequently metastasizes to cervical lymph nodes, particularly involving the central and lateral compartments. Metastasis to upper mediastinal lymph nodes is also not unusual. However, involvement of Level I cervical lymph nodes is rarely encountered. We report the case of a 25-year-old female who sought medical attention for enlarged lymph nodes in Level I of the neck and was subsequently diagnosed with papillary thyroid carcinoma based on biopsy results. Notably, ultrasound imaging revealed no detectable lesions in the thyroid gland.

## 1 Introduction

Papillary thyroid carcinoma (PTC) is the most common malignant neoplasm of the thyroid, accounting for over 80% of all thyroid cancers. It is typically characterized by indolent growth and an excellent prognosis (1). PTC commonly metastasizes to the central neck compartment as well as to lateral neck levels II, III, IV, and V (2), and involvement of upper mediastinal lymph nodes is also frequently observed (3). However, metastasis to Level I cervical lymph nodes is rare. Anatomically, Level I lymph nodes receive drainage primarily from the oral cavity, lips, nasal structures, midface, and the tip of the tongue (4), and they have no direct lymphatic connection to the thyroid gland. We report a case of thyroid papillary microcarcinoma presenting with metastatic involvement of Level I cervical lymph nodes. This report aims to raise clinical awareness of this atypical metastatic pattern in order to reduce the likelihood of misdiagnosis or oversight.

## 2 Case description

### 2.1 General information

The patient is a 25-year-old unmarried female. In 2024, she underwent a routine physical examination at a local hospital, during which thyroid nodules were identified. As she exhibited no concerning symptoms—such as pain, dysphagia, dyspnea, hoarseness, or coughing while drinking—she did not pursue further medical evaluation at that time. On April 5, 2025, she noticed a palpable mass approximately the size of a soybean in her neck. An ultrasound performed at Shaanxi Provincial People’s Hospital revealed: nodules in the left thyroid lobe (the largest measuring approximately 5.5 mm × 2.8 mm), classified as TI-RADS 3; bilateral thyroid cysts (the largest approximately 4.5 mm × 3.6 mm), classified as TI-RADS 2; and multiple abnormally enlarged lymph nodes in the cervical and anterior neck regions (the largest approximately 14.9 mm × 6.8 mm), some with calcification and signs of liquefaction. Biopsy pathology revealed no malignancy in the middle portion of the left thyroid lobe, but metastatic carcinoma was detected in lymph nodes from anterior cervical Level I and bilateral cervical Level III regions, consistent with a diagnosis of papillary thyroid carcinoma. She was diagnosed with a secondary malignant tumor of the neck and subsequently presented to our institution for further treatment.

Physical examination: the patient was in good general health and fully alert. Physical examination: On examination, the skin of the neck appeared normal in color. No obvious thyroid enlargement or nodules were palpated. Multiple enlarged lymph nodes were palpable bilaterally in the neck, the largest measuring approximately 1.5 cm × 1.4 cm. These lymph nodes were firm, smooth-surfaced, mobile, and well demarcated, with no tenderness. The patient denied any relevant family history, high-risk sexual behavior, or prior surgical procedures.

### 2.2 Laboratory and imaging examinations

**Figure 1:**
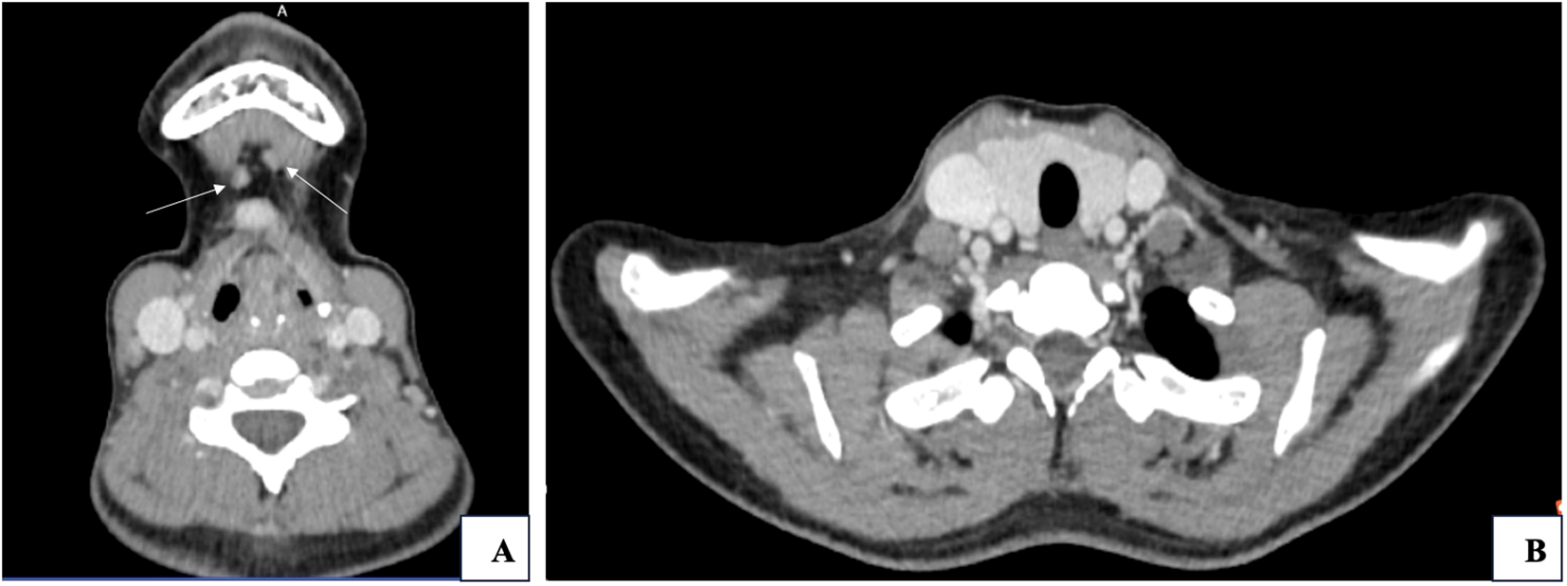
**(A)** Metastatic lymph nodes in Level I (indicated by white arrows. **(B)** Thyroid gland and surrounding structures.

Routine blood, urine, liver and kidney function tests, and electrolyte panels revealed no significant abnormalities. Parathyroid hormone: 40.34pg/mL; Thyroid function tests: FT34.66 pmol/L, FT4 20.50 pmol/L, TSH1.28μIU/mL, TG27.35ng/mL. Pathological consultation reported that fine-needle aspiration of the mid-left thyroid lobe revealed inflammatory cells but no malignant features. Conversely, cytological examination of lymph nodes from the anterior cervical Level I, left cervical, and right cervical Level III regions showed papillary thyroid carcinoma cells. Ultrasound findings included: enlarged lymph nodes in bilateral Level III and submental regions; a hypoechoic nodule above the hyoid bone, suspected to represent an enlarged lymph node; bilateral thyroid cystic nodules, categorized as TI-RADS 2. Neck CT scan (**Figure 1**) demonstrated: nodular densities in both thyroid lobes; multiple enlarged lymph nodes in the left submandibular region, mid-anterior region below the hyoid bone, and bilateral cervical Level III regions.

### 2.3 Diagnosis and differential diagnosis

Current diagnosis: Secondary malignant tumor in the neck. Multidisciplinary Team (MDT) Discussion Outcome: The primary diagnosis is most consistent with a metastatic malignant tumor of thyroid origin. Recommendation: Surgical intervention is advised, with further treatment to be based on postoperative histopathological results.

### 2.4 Treatment

**Figure 2:**
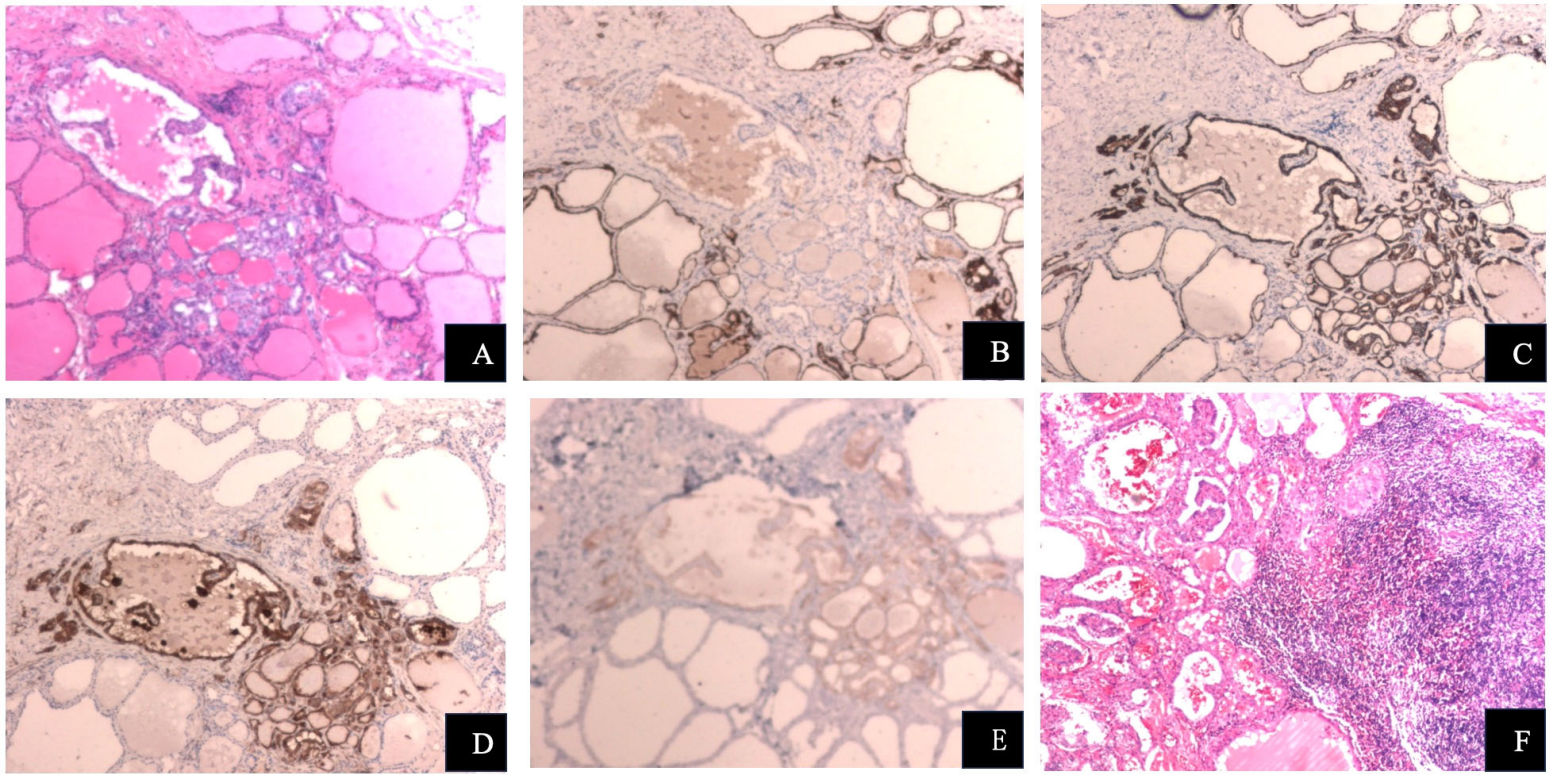
**(A)** Papillary thyroid carcinoma, HE ×400. **(B)** CD56-positive IHC, ×400. **(C)** CK19-positive IHC, ×400. **(D)** Galectin-3-positive IHC, ×400. **(E)** BRAF-positive IHC, ×400. **(F)** Level I lymph node metastasis with papillary thyroid carcinoma, HE ×400.

On April 16, 2025, the patient underwent total thyroidectomy with bilateral central neck dissection, bilateral lateral neck dissection, and Level I lymph node dissection under general anesthesia. Postoperative histopathology (**Figure 2**) revealed: Nodular goiter with a microscopic papillary carcinoma focus (approximately 1.6 mm) in the left lobe and part of the isthmus, the tumor was staged as papillary thyroid carcinoma (Stage T1aN1bM0); Nodular goiter in the right lobe and part of the isthmus. The immunohistochemistry (IHC) results are presented in **Table 1**.

**Table 1:** Immunohistochemistry (IHC) Results.

Slide Marker	CK19	Galectin-3	CD56	BRAF
Slide 17	+	+	focal +	–
Slide 13	–	–	+	–
Slide 18	/	/	/	–
Slide 9	+	–	+	–

Lymph node dissection findings: Pre-laryngeal tissue: fibroadipose and striated muscle; Pretracheal lymph node: 0/1 positive; Left central compartment lymph nodes: 0/2 positive (with thymus and parathyroid fragments); Left Level I: 1/1 positive (papillary thyroid carcinoma metastasis); Left Level IIA: 2/9 positive; Left Level IIB: 0/6 positive; Left Level III: 1/9 positive; Left Level IV: 0/11 positive; Right Level I: fibroadipose tissue; Right Level IIA: 0/3 positive; Right Level IIB: 0/3 positive; Right Level III: 3/14 positive; Right Level IV: 1/4 positive; Right Level VIA: 0/9 positive; Right Level VIB: 0/6 positive; Submental lymph nodes: 0/4 positive Additional IHC: Left Level IIA: BRAF (+), PAX8 (+), TG (+), CDX-2 (-), CK20 (-), Villin (-), TTF-1 (+), NapsinA (-), Ki-67 (5%), CK7 (+); Left Level I: P63 (-), P53 (1+), CK5/6 (-), Ki-67 (5%).

### 2.5 Treatment outcomes, follow-up, and prognosis

Postoperative thyroid function test results (2025.05.16):FT3 4.77 pmol/L, FT4 19.57 pmol/L, TSH 0.219 μIU/mL, TG 0.837 ng/mL (preoperative level was 27.35 ng/mL, showing a significant decrease compared to preoperative values).Following discharge, the patient reported no significant discomfort and adhered to medical advice by attending the Department of Nuclear Medicine for iodine-131 treatment one month later. The patient expressed satisfaction with both the diagnosis and treatment. The diagnostic workup was conclusive, the therapeutic outcome was excellent, and postoperative recovery has progressed well without any current complaints.

## 3 Discussion

Metastasis of papillary thyroid carcinoma to level I cervical lymph nodes is exceedingly rare, with only a few reported cases. Level I lymph nodes include the submental (IA) and submandibular (IB) groups, which primarily drain lymph from the oral cavity, lips, nasal region, midface, and anterior tongue. In contrast, the thyroid gland predominantly drains into the central compartment (level VI) and lateral neck compartments (levels II–V) (5), with no direct anatomical connection to level I. This anatomical separation explains why thyroid cancer seldom metastasizes to level I. When such metastasis does occur, it may result from direct invasion of the floor of the mouth or base of the tongue by the primary lesion, allowing for local spread to level I, or from altered lymphatic drainage due to obstruction in central or lateral cervical lymph nodes. Nevertheless, such cases are highly uncommon. Previous studies have shown (6) that lymph node metastasis in thyroid carcinoma predominantly involves the central compartment (level VI, with a metastasis rate of approximately 20%–60%) and lateral compartments (levels II–V), whereas metastasis to level I occurs in less than 1% of cases. For example, in a study on differentiated thyroid carcinoma involving 6,027 patients, metastasis rates were reported as 36.05% for level VI and 20.41% for levels II–V, with no explicit mention of level I involvement (7). These data suggest that level I metastasis is an unusual phenomenon, potentially indicating a more aggressive tumor phenotype or the presence of atypical lymphatic drainage pathways.

Ultrasound is the primary modality for evaluating cervical lymph nodes in thyroid cancer (8); however, its sensitivity in detecting level I lesions is limited. The superficial location and small size of level I lymph nodes make them easily mistaken for the submandibular gland or surrounding musculature. Additionally, benign conditions such as reactive hyperplasia secondary to oral infection can complicate diagnostic interpretation. Neck CT, especially contrast-enhanced CT, is instrumental in identifying suspiciously enlarged lymph nodes. Fine-needle aspiration cytology combined with thyroglobulin measurement in needle washout fluid (9) can further clarify the nature of enlarged nodes and assist in guiding clinical management.

Current guidelines from the American Association of Endocrine Surgeons do not recommend routine dissection of level I lymph nodes in thyroid carcinoma (10). Instead, expanded dissection is advised only when there is direct tumor invasion of adjacent anatomical structures (e.g., the oral floor) or when imaging strongly suggests metastasis. When level I involvement is identified, dissection of both central and lateral compartments is necessary. In this case, preoperative biopsy confirmed papillary carcinoma metastasis in level I lymph nodes. The patient subsequently underwent total thyroidectomy along with bilateral central, lateral neck, and level I lymph node dissection. Postoperative recovery was uneventful, and the patient is preparing for radioactive iodine therapy.

The patient initially presented with enlarged cervical lymph nodes. Preoperative ultrasound showed benign thyroid nodules, and no primary lesion was identified intraoperatively via frozen section, leading to a provisional diagnosis of occult thyroid carcinoma (11). However, after extensive pathological examination of the postoperative thyroid tissue, a minute primary lesion measuring 1.6 mm in diameter was discovered. Pathologists should remain vigilant and conduct meticulous sampling and examination in such cases, particularly when preoperative imaging does not reveal a primary lesion despite confirmed lymph node metastasis. Careful evaluation is essential to avoid misdiagnosis as occult thyroid carcinoma.

This case involves a rare metastatic site of thyroid carcinoma, which is uncommon in clinical practice. However, there were still certain limitations in the diagnostic and treatment process. First, regarding diagnosis: preoperative fine-needle aspiration did not include testing for the BRAF V600E mutation, and the preoperative evaluation relied solely on ultrasound findings from an outside hospital, without repeat imaging at our hospital. Second, in terms of follow-up: the duration since the case occurred has been relatively short, and long-term follow-up data are currently lacking. We will continue tracking the patient’s progress over time.

## 4 Conclusion

Metastasis of thyroid carcinoma to level I cervical lymph nodes is rare due to anatomical constraints. Its occurrence may signal altered tumor biology and warrants increased clinical awareness. Future research should focus on uncovering the molecular mechanisms and lymphatic anomalies associated with level I metastasis to improve patient outcomes.

## Data Availability

The raw data supporting the conclusions of this article will be made available by the authors, without undue reservation.
